# How Does Our Motor System Determine Its Learning Rate?

**DOI:** 10.1371/journal.pone.0049373

**Published:** 2012-11-12

**Authors:** Robert J. van Beers

**Affiliations:** 1 MOVE Research Institute Amsterdam, Faculty of Human Movement Sciences, VU University Amsterdam, Amsterdam, The Netherlands; 2 Department Physics of Man, Helmholtz Institute, Utrecht University, Utrecht, The Netherlands; Bielefeld University, Germany

## Abstract

Motor learning is driven by movement errors. The speed of learning can be quantified by the learning rate, which is the proportion of an error that is corrected for in the planning of the next movement. Previous studies have shown that the learning rate depends on the reliability of the error signal and on the uncertainty of the motor system’s own state. These dependences are in agreement with the predictions of the Kalman filter, which is a state estimator that can be used to determine the optimal learning rate for each movement such that the expected movement error is minimized. Here we test whether not only the average behaviour is optimal, as the previous studies showed, but if the learning rate is chosen optimally in every individual movement. Subjects made repeated movements to visual targets with their unseen hand. They received visual feedback about their endpoint error immediately after each movement. The reliability of these error-signals was varied across three conditions. The results are inconsistent with the predictions of the Kalman filter because correction for large errors in the beginning of a series of movements to a fixed target was not as fast as predicted and the learning rates for the extent and the direction of the movements did not differ in the way predicted by the Kalman filter. Instead, a simpler model that uses the same learning rate for all movements with the same error-signal reliability can explain the data. We conclude that our brain does not apply state estimation to determine the optimal planning correction for every individual movement, but it employs a simpler strategy of using a fixed learning rate for all movements with the same level of error-signal reliability.

## Introduction

Over the last few decades, many studies have examined whether optimality principles can explain human motor behaviour. Although different frameworks have been used, such as optimal (feedback) control (for reviews, see: [1], [2]), statistical decision theory (for a review, see: [3]), and Bayesian decision theory (for reviews, see: [4], [5]), all of these studies found evidence for the principle that our motor system attempts to minimize a cost function that includes variability or uncertainty, among others (see also: [6], [7]). The optimality approach has extended our understanding of motor control enormously, but it raises the question *to what extent* motor control is optimal. Is every individual movement that we make optimal, or is only some average behaviour optimal?

We will address this question for the example of determining the learning rate in motor learning. When we produce a movement error, this error can be used to improve planning of future movements. The learning rate is the proportion of the error by which planning is corrected. The learning rate does not need to be constant but could depend on factors such as the reliability of the error signal and the uncertainty of the motor system’s own state. The problem of determining the learning rate has similarities with the engineering problem of estimating the state of a system through noisy observations. Every new observation can be used to improve the state estimate, but the extent by which the estimate should be adjusted depends on the reliability of the new observation and on the uncertainty of the previous state estimate. The more reliable the observation and the more uncertain the previous state estimate, the larger the adjustment should be. Under certain conditions, such as that the system dynamics are linear and known and the noise is white and Gaussian, the Kalman filter [8] is the optimal state estimator, as it is the unbiased estimator with the lowest variance. It has therefore been proposed [9] that the motor system could use a Kalman filter to determine its learning rate. The optimal learning rate then equals the Kalman gain. Three studies [10]–[12] found support for this hypothesis, as it was observed that the learning rate increases when the error-signal reliability or the uncertainty of the state estimate is increased. These findings were obtained by averaging estimated learning rates over large numbers of trials.

If the motor system uses a Kalman filter to determine its learning rate, the learning rate would not only be optimal on average, but it would be optimal in every individual movement. The aim of this study is to determine whether this is the case. The standard way to estimate learning rates is to use perturbations that disturb the motor performance, and to analyze how motor planning changes in response to induced errors. However, subjects in this paradigm face a dual task as they should both estimate the source of each error and determine an appropriate correction [13], [14]. For self-generated errors resulting from inaccurate motor planning, a large correction would be appropriate, but for errors that have an external origin such as an incidental gust of wind, no correction should be made. Since a subject’s estimate of the error source may vary from trial to trial, it is difficult to obtain accurate estimates of the learning rate in this paradigm. We therefore used a method that did not involve perturbations, so that all errors were self-generated. Subjects simply made a series of reaching movements to a fixed target. They could not see their hand during the movement, but they received visual feedback about their error immediately after each movement. This allowed them to translate the observed error into a planning correction for the next movement. There were three different levels of error-signal reliability.

It is not possible to obtain reliable estimates of the learning rate for individual movements because effects of motor noise cannot be distinguished from planning corrections in individual movements. It is nevertheless possible to test whether subjects used a Kalman filter for every individual movement in a series to the same target, as the Kalman filter makes specific predictions for the serial correlations of movement endpoints, and for how the mean squared movement error will evolve during a series.

We will first present the results of the experiment. We will then show how the Kalman filter can be used to make optimal planning corrections in this paradigm. Next, we will demonstrate that the observed behaviour is not consistent with the predictions of the Kalman filter. Finally, we will show that the data can be explained by a simpler model in which the learning rate is fixed during a session, but varies with the error-signal reliability.

## Results

**Figure 1:**
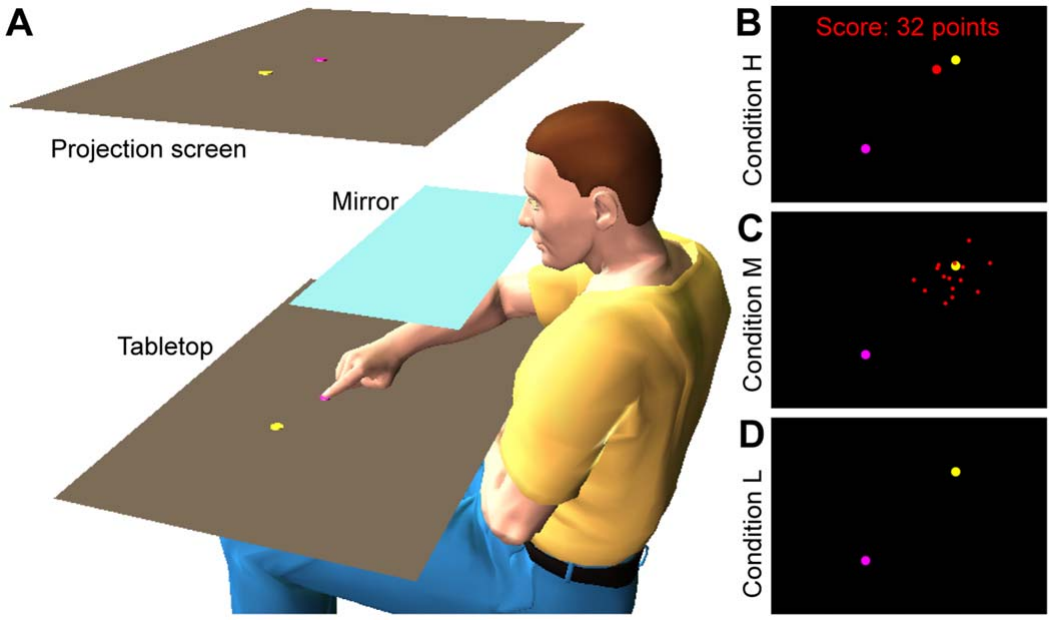
Experimental setup and error-signals in each condition. A Subjects were seated at a table, and had no direct vision of the table and their arm because that was blocked by a black cloth (not shown) and a mirror that was placed midway between the tabletop and a projection screen. An LCD projector (not shown) projected images onto this screen. When the subject looked in the mirror, he saw the images at the location of the tabletop. In the shown situation, the subject just started the movement from the start position (pink disc) to the target (yellow disc). **B** In condition H (high error reliability), a red disc was shown at the movement endpoint immediately after the movement end was detected. In addition, a score was displayed that decreased with the distance from the endpoint to the target. **C** In condition M (medium error reliability), a cloud of 15 dots, drawn from a Gaussian centred on the actual endpoint, was shown immediately after the movement. **D** In condition L (low error reliability), subjects received no visual feedback about their movement endpoints.

Subjects made 30 successive movements to the same target in each series. One session consisted of 24 series of the same condition, each with a different target. All targets were located at 10 cm distance from a fixed start location, in equally spaced directions. Subjects could not see their hand during the movement, but they received visual information about the movement endpoint immediately after each movement (see Fig. 1A for the setup). The visual endpoint feedback depended on the experimental condition. In condition H (high error reliability), a small red disc was shown exactly at the actual endpoint location (Fig. 1B). In condition M (medium error reliability), subjects saw a cloud of 15 red circular dots, drawn from a circular Gaussian distribution centred on the actual endpoints (Fig. 1C). Subjects received no visual information about their endpoints in condition L (low error reliability, Fig. 1D).

### Observed Error Correction

**Figure 2:**
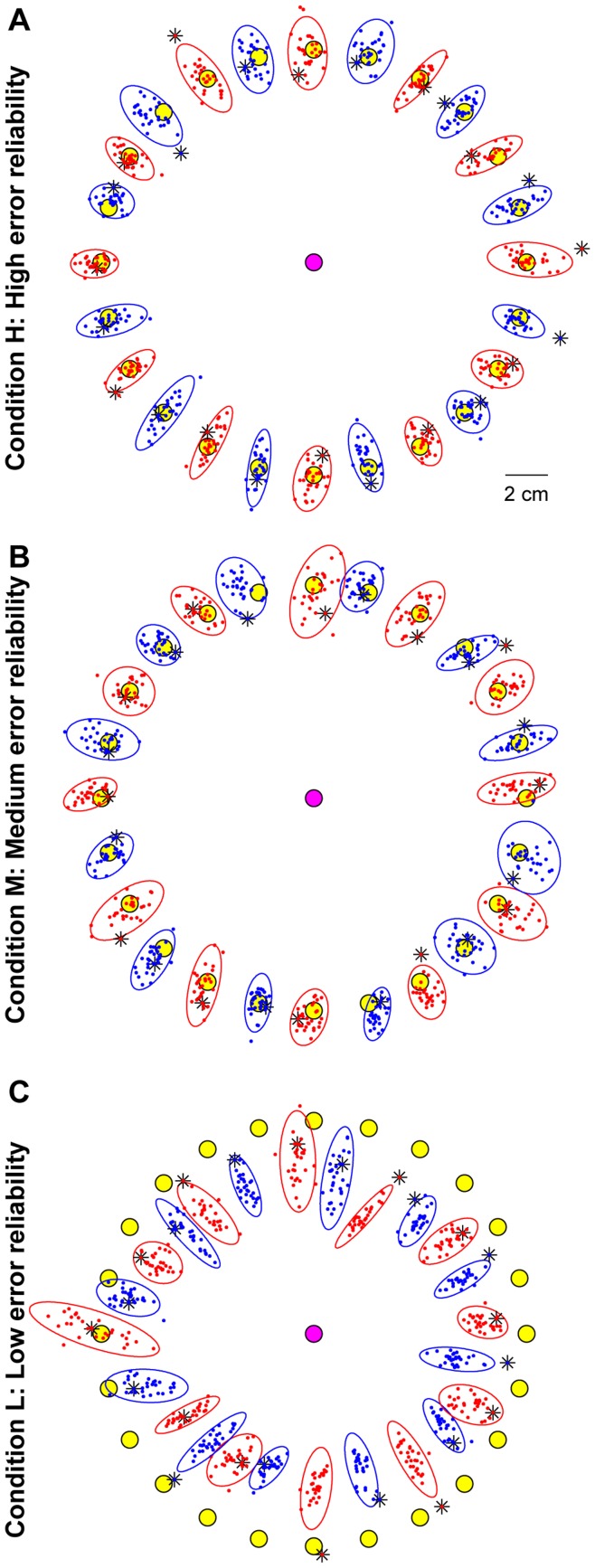
Examples of endpoints in each condition. The start position (pink disc), the targets (yellow discs), all the endpoints (small dots) and their 95% confidence ellipses of a representative subject (SG). Red and blue colours are used for the endpoints for different targets in alternating order. Asterisks denote the endpoints of the first movement to a target. **A** Condition H. **B** Condition M. **C** Condition L.

Figure 2A shows all the endpoints of a representative subject in condition H. Three effects stand out, and these were found for all subjects. First, movements were on average quite accurate. Second, the endpoint variability was anisotropic. Variability in the Extent component (the component parallel to the vector from the start location to the mean endpoint of the series) was generally larger than that in the Direction component (the component orthogonal to the Extent component), which is consistent with earlier observations [15], [16]. Third, the endpoint of the first movement to a target (marked by asterisks) often differed substantially from later endpoints.

The overall picture in condition M (Fig. 2B) is quite similar to that in condition H. All three points mentioned above apply also to this condition. The last two apply also to condition L (Fig. 2C), but the first point does not apply here as movements were often systematically biased. The subject whose endpoints are shown in Figure 2 tended to systematically undershoot the targets, but this was not observed for all subjects. Some consistently undershot the targets, others produced mainly overshoots, but for the majority the pattern was more complicated, with overshooting for some targets and undershooting for others. In addition, all subjects displayed directional errors for some targets. The endpoint variance (the sum of the variance of the Extent and Direction components) did not significantly differ between conditions (repeated measures ANOVA: *F*_(2,14)_ = 3.21; *p* = 0.07). The mean endpoint variance was 86 mm^2^; the mean variances for the Extent and Direction components were 67 mm^2^ and 18 mm^2^, respectively.

In all conditions, the endpoint of the first movement to a target often differed considerably from later ones. This suggests that planning of the first movement to a target was often inaccurate. The fact that only the first endpoint differed implies that the error in this movement was used to adjust planning of the next movement. In conditions H and M, the error signal was visual, and it was reliable enough to reduce the error. In condition L, the error signal arose by comparing the felt finger location to the seen target position. Idiosyncratic biases in the proprioceptive sense of finger location relative to visual targets [17], [18] will have caused the sizeable and subject-dependent constant errors in this condition. Remarkably, for the subject whose data are shown in Figure 2C, the first movement to a target was often quite accurate, whereas all later ones were less accurate. This was observed for more subjects. Apparently, motor planning was initially relatively accurate, but the proprioceptive-visual bias was so large that later movements had to be less accurate to give subjects the feeling that they hit the target.

**Figure 3:**
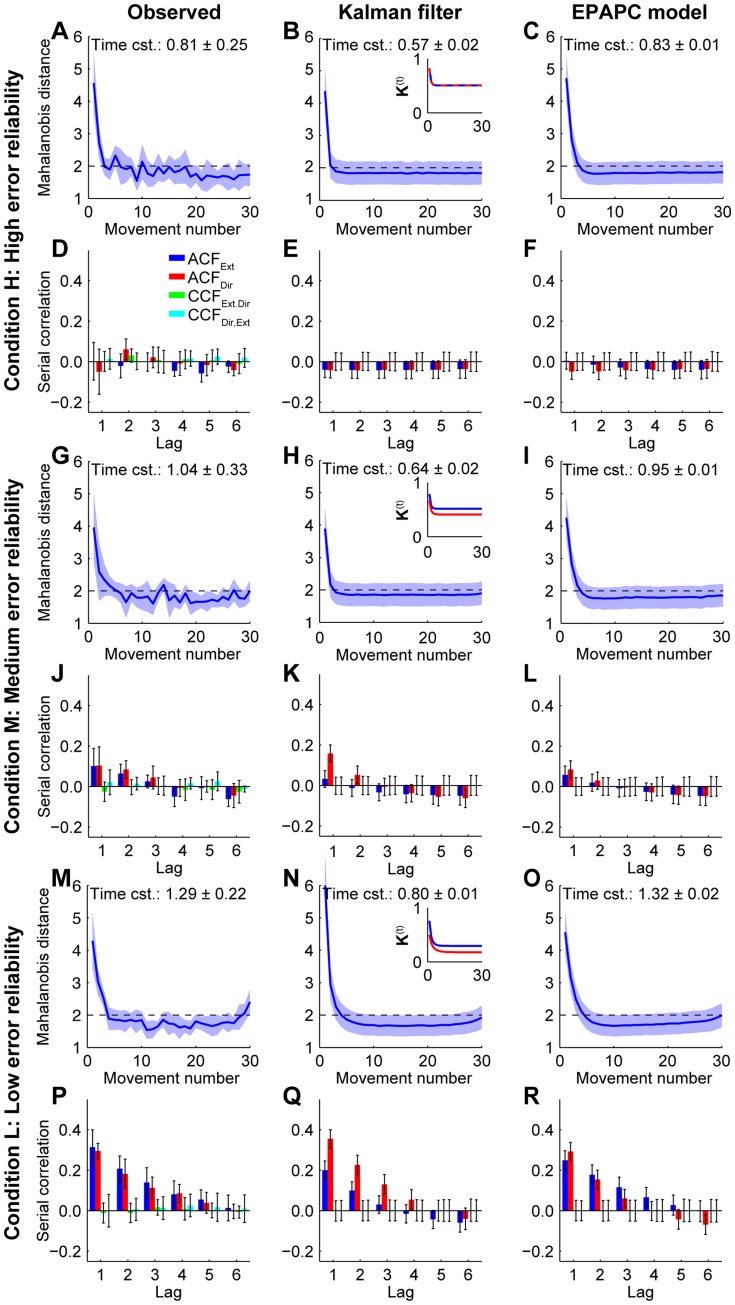
Observed and predicted learning curves and serial correlations for each condition. **A** Observed learning curve in condition H. The shaded area represents the between-subject standard deviation. The dashed line at 2 shows the expected value if all endpoints are drawn independently from an identical Gaussian. **B** Learning curve in condition H as predicted by the Kalman filter. The shaded area indicates the between-subjects standard deviation, as predicted by this model. Inset: Kalman gain as a function of trial number. Blue: Extent component, red: Direction component. **C** Learning curve in condition H as predicted by the EPAPC model. **D** Observed serial correlations in condition H. Error bars denote the between-subject standard deviation. ‘Ext’ and ‘Dir’ refer to the Extent and Direction component, respectively. **E** Serial correlations in condition H as predicted by the Kalman filter. Error bars denote the between-subjects standard deviation, as predicted by this model. **F** Serial correlations in condition H as predicted by the EPAPC model. **G** *–* **L**, The same as in **A** *–* **F**, but now for condition M. **M** *–* **R**, The same as in **A** *–* **F**, but now for condition L.

**Figure 4:**
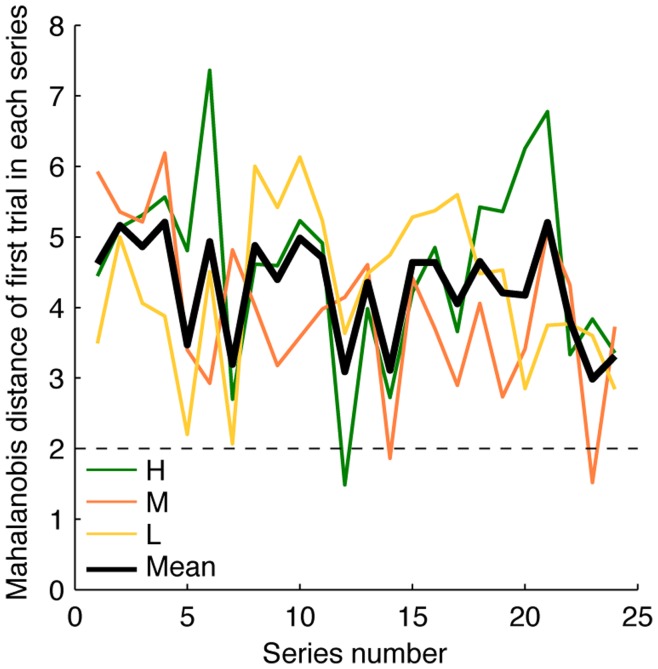
Mahalanobis distance of the first trial in a series as a function of the series number. Colored lines represent the average (across all subjects) per condition, whereas the black line denotes the mean of all conditions. The dashed line at 2 shows the expected value if the endpoint in the first trial does not, on average, differ more from the mean endpoint than the endpoints in later trials.

Learning curves were constructed to quantify how quickly subjects shifted their endpoints in the beginning of a series towards the steady-state position. We calculated the Mahalanobis distance (“the squared number of standard deviations that an endpoint differs from the mean endpoint of the series”, see Methods) for every endpoint, and plotted the mean Mahalanobis distance as a function of the movement number in the series. Figure 3A shows that subjects corrected their initial errors in condition H in a couple of movements. Fitting exponentials to the learning curves produced an estimated time constant of these corrections of 0.81±0.25 movements (weighted average across subjects ±95% confidence interval). Figure 4 shows that the large initial errors were not restricted to the first series in a session, but they occurred in all series of a session.

**Figure 5:**
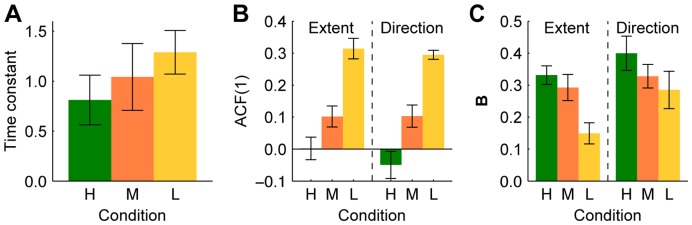
Time constants, lag 1 autocorrelations and learning rates for each condition. **A** Weighted average (across all subjects) of the estimated time constants, with error bars representing 95% confidence intervals. **B** Mean ACF(1) of the Extent and Direction components. Error bars represent the standard error in the mean. **C** Mean learning rates for the Extent and Direction components, as determined by fitting the EPAPC model. Error bars represent the standard error in the mean.

In conditions M and L, subjects also changed their endpoints in a couple of movements (Figs. 3G, M), but the rate at which this occurred decreased with increasing error uncertainty (Fig. 5A). A repeated measures ANOVA in which the time constants of individual subjects were weighted with the inverse of the squared width of their confidence interval confirmed that the time constants varied significantly between conditions (*F*_(2,35)_ = 15.32; *p*<0.0001). Another, subtle, difference between the learning curves is that the end is as good as flat in conditions H and M, whereas it rises in condition L. In all conditions, initial movements could be inaccurate in all series of a session (Fig. 4).

The observed serial correlations are plotted in Figures 3D,J,P. We will first focus on the lag 1 autocorrelations (ACF(1)s), which express the statistical relationship between the endpoints of consecutive movements. The ACF(1) is positive when the endpoints of consecutive movements tend to be close together relative to the overall variability, whereas it is negative when they tend to be far apart, on opposite sides of the mean endpoint. It is zero when consecutive endpoints are statistically independent of one another. The ACF(1) of both the Extent and Direction components was close to zero in condition H (Fig. 3D). It is not possible to test whether they were significantly different from zero because the estimation of autocorrelations from short time series is fundamentally biased [19], [20]. Both ACF(1)s are about 0.1 in condition M, and about 0.3 in condition L (see also Fig. 5B). A 3 (conditions: H, M, L)×2 (component: Extent, Direction) repeated measures ANOVA showed that the ACF(1) varied between conditions (*F*_(2,14)_ = 56.72; *p*<0.00001) but not between components (*F*_(1,14)_ = 0.45; *p*>0.5), and there was no significant interaction between condition and component (*F*_(2,14)_ = 0.91; *p* = 0.43). Post-hoc Tukey’s honestly significant difference tests showed that the autocorrelation differed significantly between any pair of conditions (all *p*≤0.001). The lag 1 cross-correlations, which express the statistical relation between the Extent component of one endpoint and the Direction component of the previous or next endpoint, are approximately zero in all conditions. This suggests that in all conditions, an error in one component did not lead to a corrective adjustment in the planning of the other component.

**Figure 6:**
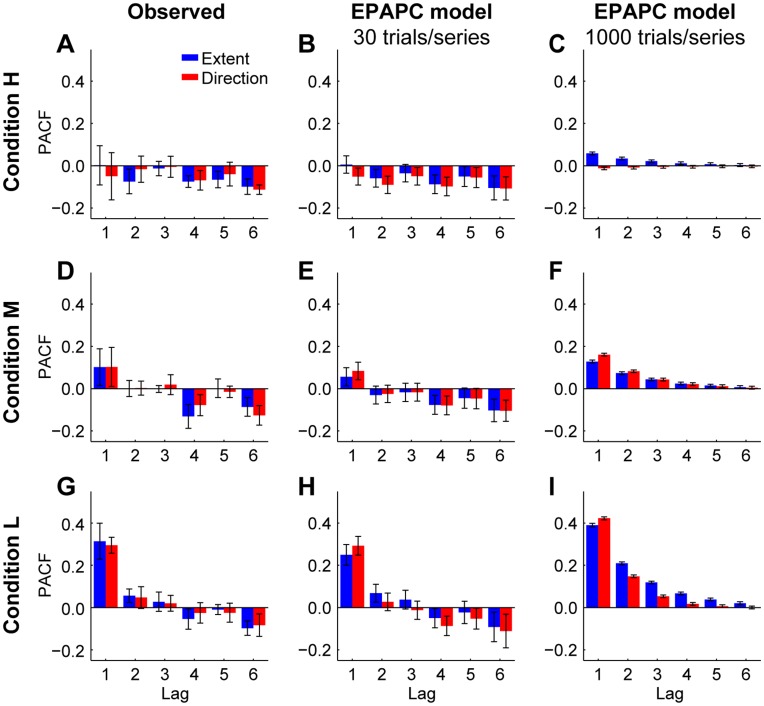
Partial autocorrelation functions (PACFs). **A** Mean PACFs observed in condition H. Error bars denote the across-subjects standard deviation. **B** PACFs predicted by the EPAPC model for series of 30 movements in condition H. Error bars denote the across-subjects standard deviation as predicted by the model. **C** PACFs predicted by the EPAPC model for series of 1000 movements in condition H. **D**–**F** Same as **A**–**C** but now for condition M. **G**–**I** Same as **A**–**C** but now for condition L.

In condition L, the autocorrelations at several lags greater than 1 are also positive (Fig. 3P). These correlations express the statistical relationship between the endpoints of movements that did not follow each other directly, but where one or more movements were made in between. However, these positive values reflect not only the genuine correlations between these endpoints, but also the linear dependence on the endpoints of the intervening movements. The partial autocorrelation function at lag *k* (PACF(*k*)) is the autocorrelation between the endpoints of movements *t* and *t – k* after their linear dependence on the intervening endpoints has been removed. Partial autocorrelations of the observed endpoints are shown in Figures 6A, D, G. The most striking difference with the autocorrelations in Figure 3D, J, P is that the partial autocorrelations decrease more rapidly, and are close to zero already at lag 2. The negative PACFs for lags above 3 are an artefact of using short time series (see section EPAPC model: predictions).

In summary, we found that both the time constant of the learning curve and the lag 1 autocorrelation of the endpoints increased with increasing error-signal uncertainty. Since the time constant and the autocorrelation increase when smaller error corrections are made, these results confirm the earlier finding [11], [12] that learning slows down when the error-signal reliability is decreased.

### Kalman Filter: Model

We used the Kalman filter to determine the optimal planning correction for individual movements. The task of motor planning is to generate motor commands that will bring the finger to the target. The substantial errors in the first movement to a target (Figure 2) indicate that this is not a trivial task. Observed movement errors are therefore used to improve motor planning. The central idea of using the Kalman filter for this process is that the brain estimates the endpoint that will result from a planned motor command. This estimate is updated after observing the actual endpoint and this updated estimate is then used to improve planning of the next movement. It is therefore important to distinguish the actual movement planning and execution signals in the subject’s nervous system from the brain’s estimates of their resulting endpoints. First consider the actual movement planning and execution signals.

Let
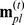
be the endpoint that would result if the centrally planned motor command of movement *t* would directly drive the movement without being corrupted by noise. We will refer to this as the *planned aim point* [21]. Actual motor commands are however corrupted by noise in the relay of the motor command by motoneurons and in the conversion into mechanical forces in muscles [16], [22], [23]. We will refer to this as *execution noise*. Its consequence is that the actual endpoint **x** ^(*t*)^ will differ from the planned aim point:
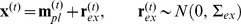
(1)


where 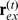 is a random vector that represents the effect of execution noise, which is drawn from a zero-mean Gaussian with covariance matrix Σ*_ex_*. We assume that the sensed endpoint 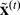 is a read-out of the actual endpoint that is corrupted by sensory noise:
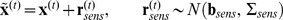
(2)


where 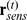 is a random vector that represents the effect of sensory noise. It is drawn from a Gaussian with mean **b** *_sens_* that can be non-zero to account for the possibility that sensed errors are biased, such as in condition L (this is not a problem for the Kalman filter described below; it just means that the mean endpoint will be biased by an amount −**b** *_sens_*).

We next assume that the planned motor command of the movement just executed will serve as a basis for the planning of the next movement, while a (yet to be determined) planning correction **c** ^(*t*)^ is added based on the observed error in the previous movement. Since the generation of the new motor command is a stochastic process [24], the effect of *planning noise* 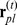 is added as well:
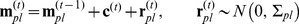
(3)Planning, execution and sensory noise are assumed to be white and independent of one another.

The actual endpoint is unknown to the subject. It can be eliminated from the above equations to yield:
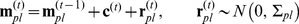
(4a)

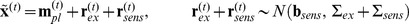
(4b)


The first equation can be viewed as a state equation with state 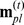, and the second equation is an output equation.

We will now use the Kalman filter to determine the planning corrections **c** ^(*t*)^. The Kalman filter recursively estimates the planned aim points 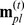 by optimally combining predictions and observations. The Kalman filter’s time update equations give the a priori (or predicted) planned aim point and the a priori error covariance matrix **P** ^(*t*)^ when the new movement has been planned (with known correction) but before its endpoint is observed:
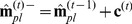
(5a)

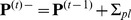
(5b)


Hats denote estimates and the minus symbol indicates that these are a priori values. The measurement update equations give the a posteriori values that are obtained after the endpoint is observed:
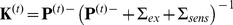
(6a)

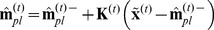
(6b)

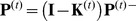
(6c)


Here, **K** ^(*t*)^ is the Kalman gain that optimally weights the observed endpoint relative to the a priori estimate, 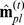 is the estimate of the planned aim point in movement *t* after its endpoint has been observed, **I** is the identity matrix, and −1 denotes the matrix inverse.

The planning correction for the next movement should correct for the difference between the estimated planned aim point and the target location **x** *_T_*:
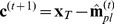
(7)


When we substitute this expression in the first time update equation (Eq. 5a), we obtain:
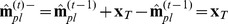
(8)


This equation shows that for this planning correction, every movement is planned such that it is expected to be accurate. The second measurement update equation (Eq. 6b) then becomes:
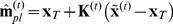
(9)


This equation shows that after the endpoint has been observed, the estimated planned aim point of the movement just executed is corrected by an amount that is proportional to the sensed error (the difference between the sensed endpoint and the target location). When we substitute this into Eq. 7, we find that the planning correction is equal to:
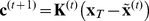
(10)


The planning correction is thus proportional to the sensed error, and the Kalman gain **K** ^(*t*)^ acts as the learning rate that determines the proportion of the error that is corrected for. When we combine all the results, we can rewrite the equations for the actual movement planning and execution (Eq. 4) as:
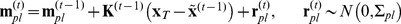
(11a)

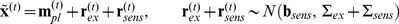
(11b)where the Kalman gain is determined recursively according to equations 5 and 6.

To complete the specification of the Kalman filter, we have to choose the initial values of the state estimate and its error covariance. The planned aim point of the first movement in a series depends on the initial state estimate. The fact that the first movement could be quite inaccurate in each series (Figs. 2 and 4) suggests that the initial state estimate had a relatively large uncertainty. We therefore assumed that the Kalman filter was reset at the start of each new series. We modelled the initial error covariance as:
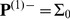
(12)where Σ_0_ is a covariance matrix, that, in order to produce relatively large errors, has elements that exceed those of Σ*_pl_* and Σ*_ex_*. Since there was no systematic pattern in the direction of the initial errors (they could be undershoots and overshoots, and the direction could be off in either direction), we initialized the state estimate as:



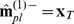
(13)As a result, the first planned aim point was:
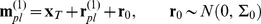
(14)where **r**_0_ is a random vector that reflects the uncertainty of the initial state estimate. As a result of this large initial uncertainty, the Kalman gain, and therefore the learning rate, will initially be large and then decrease to stabilize at a lower value.

The model assumes that the dynamics of the system are linear, that all noise is Gaussian, and that planning corrections are proportional to the sensed error. Previous studies [10], [25] have shown that the assumptions of linearity and Gaussian noise capture the trial-by-trial behaviour in repeated reaching movements very well, and that including nonlinearities or deviations from normality does not lead to improvements in explaining observed reaching behaviour. The assumption of proportional planning corrections is also reasonable because errors in the present study were generally smaller than 2 cm, and for errors of this size, corrections have been shown to be proportional to errors [14].

### Kalman Filter: Predictions

We tested whether the Kalman filter can explain the data by evaluating whether it can reproduce the observed learning curves and autocorrelations. The predictions depend on the various covariance matrices defined above. Since it is not possible to obtain accurate estimates of all of these matrices, we followed a different approach in which we essentially determined whether any set of values of the covariance matrices could reproduce both the observed learning curves and the autocorrelations. To reduce the number of free parameters, we assumed that all covariance matrices were diagonal (this is justified by the observation that all endpoint ellipses had their major axis roughly aligned with the movement direction), we used literature values or estimates obtained in a control experiment for the error-uncertainty covariance matrix Σ*_sens_* (see Methods for details), and we assumed that all the other covariance matrices (Σ*_pl_*, Σ*_ex_* and Σ_0_) differed from one another by a scaling factor. This assumption was motivated by the finding of Cheng and Sabes [25] who estimated matrices corresponding to Σ*_pl_* and Σ*_ex_* directly from data, and found that both were anisotropic with a larger variance in the Extent than in the Direction component. Specifically, we parameterized these matrices as: Σ*_pl_* =  *sw*Σ*_mot_*, Σ*_ex_* =  *s*(1– *w*)Σ*_mot_* and: Σ_0_ =  *cs*Σ*_mot_*, where Σ*_mot_* =  [4 0; 0 1] mm^2^ is a prototype covariance matrix to which Σ*_pl_*, Σ*_ex_* and Σ_0_ are proportional. Parameter *s* scales all these matrices relative to Σ*_sens_*, *w* determines the relative size of the planning and execution covariance matrices, and *c* scales the initial state-estimate uncertainty. We assumed that the (1, 1) element of Σ*_mot_* was 4 times as large as the (2, 2) element because the ratio of Extent to Direction variance was about 4 for all subjects and in all conditions. Hence, there were three free parameters: *s*, *w* and *c*. For condition H, we assumed that the error-signal uncertainty was negligible (Σ*_sens_* = **0**). Since both the Mahalanobis distance and the autocorrelation are standardized values that are independent of the magnitude of the endpoint variability, the predictions were independent of the value of *s* for this condition. As a result, there were only two free parameters (*w* and *c*) for this condition.

For each subject and each condition, we determined the values of the free parameters that minimized the difference between the predicted and observed values of the initial value of the learning curve, the learning-curve time constant, and the lag 1 and lag 2 autocorrelations of the Extent and Direction component (see Methods for details). Table 1 shows the means (of all subjects) and standard errors of the best parameter estimates. The second column of Figure 3 shows the predictions of the Kalman filter for these parameter values. The model reproduces the increase of the time constant and the ACF(1)s with decreasing error-signal reliability. However, the Kalman filter corrects faster for the initial error than the subjects did, as the time constant predicted by the Kalman filter was for each condition significantly shorter than that estimated from the data (0.57 vs. 0.81, 0.65 vs. 1.04, and 0.80 vs. 1.29 movements for conditions H, M and L, respectively; all *p*<0.05, two-tailed weighted *t* tests: [26]). This suggests that the actual corrections early in the series were smaller than those generated by the Kalman filter. This could be related to the fact that the Kalman filter initially has a large learning rate (Kalman gain), which later decreases (see insets in Figs. 3B, H, N; where the Kalman gain is plotted separately for the Extent and Direction components).

**Table 1:** Best parameter estimates (means of all subjects ± s.e.m.) for the Kalman filter.

Condition	*w*	*s*	*c*
H	0.35±0.09	–	2.75±0.33
M	0.45±0.10	17.6±0.7	2.57±0.49
L	0.32±0.08	8.3±3.5	7.84±2.15

The serial correlations predicted for condition H (Fig. 3E) agree well with the observed ones (Fig. 3D). However, for conditions M and L, the Kalman filter predicts that the ACF(1) is larger for the Direction than for the Extent component (see Figs. 3K, Q). This is a consequence of the fact that the planning and execution noise covariance matrices are anisotropic, with a larger variance in the Extent than in the Direction component, whereas the sensory noise covariance matrices are isotropic (see Methods). As a result, the ratio of measurement to process noise variance is larger for the Direction than for the Extent component. This leads to a larger Kalman gain for the Extent component (see insets of Figs. 3H, N), which, in turn, leads to a smaller autocorrelation. Such a difference was however not found in the data (see Figs. 3J, P).

In summary, the Kalman filter predicts faster correction for initial errors than observed and it predicts different autocorrelations for the Extent and Direction component whereas the observed ones do not differ. We conducted a sensitivity analysis to examine whether these failures of the model can be the result of incorrect assumptions in the parameterization of the covariance matrices. In this analysis we repeated the analysis above several times, where each time the value of one or two parameters was doubled or halved. The parameters that were varied were: Σ*_mot_*, the aspect ratio of Σ*_mot_* (the diagonal elements were varied such that the ratio of the two diagonal elements was doubled or halved, while their sum remained the same), Σ*_sens_*, the aspect ratio of Σ*_sens_*, while we also varied the aspect ratios of Σ*_pl_* and Σ*_ex_* simultaneously such that one aspect ratio was doubled while the other was halved. We also considered the case that Σ*_pl_* was isotropic; here, we assumed that Σ*_pl_* =  *sw* **I**, with **I** the identity matrix, whereas Σ*_ex_* =  *s*(1– *w*)Σ*_mot_*, as before. The variations of Σ*_sens_* were not applicable to condition H because we assumed Σ*_sens_* = **0** for this condition. We therefore examined the effect of Σ*_sens_* being non-zero for this condition.

Table 2 shows the results of the sensitivity analysis for condition H. Both the parameter estimates and the corresponding time constant and ACF(1)s are shown. This table shows that large changes of Σ*_mot_*, Σ*_pl_* and Σ*_ex_* have very little effect on the time constants and ACF(1)s, and the predicted time constant is always well below the observed one. The only parameter change that leads to a time constant near the observed one is assuming a non-zero Σ*_sens_* (last row of Table 2). However, the error-signal uncertainty required for this (Σ*_sens_* = 0.2Tr(Σ*_mot_*)**I**) is unrealistically large: To obtain an endpoint variance matching the data (86 mm^2^), we would need *s* ≈ 14, which corresponds to Σ*_sens_* ≈ [14 0; 0 14] mm^2^. In other words, the standard deviation in the perceived size of an error of 7 mm (a typical error) would be almost 4 mm. This is unrealistically large as subjects saw the target and the endpoint simultaneously (see Fig. 1B).

**Table 2:** Results of the sensitivity analysis for the Kalman filter for condition H.

	*w*	*c*	Time cst.	ACF(1)Ext	ACF(1)Dir
Observed			0.81	0.002	−0.050
Baseline	0.35	2.75	0.56	−0.042	−0.043
Σ*_mot_* halved	0.35	2.75	0.57	−0.042	−0.043
Σ*_mot_* doubled	0.35	2.75	0.56	−0.042	−0.044
AR(Σ*_mot_*) halved	0.35	2.75	0.57	−0.042	−0.040
AR(Σ*_mot_*) doubled	0.35	2.75	0.56	−0.041	−0.042
AR(Σ*_pl_*) halved AR(Σ*_ex_*) doubled	0.39	2.63	0.54	−0.042	−0.043
AR(Σ*_pl_*) doubled AR(Σ*_ex_*) halved	0.30	2.94	0.59	−0.041	−0.041
Σ*_pl_* isotropic	0.40	2.29	0.59	−0.042	−0.042
Σ*_sens_* = 0.2Tr(Σ*_mot_*).**I**	0.08	2.96	0.77	−0.010	0.044

AR: aspect ratio.

For all of these simulations, we assumed *s* = 1.

The sensitivity analysis for condition M (Table 3) shows that changing the parameters cannot solve the problems that the predicted time constant is too short and that the predicted ACF(1)s for the two components are different. This is also the case for most parameters for condition L (Table 4), although it is possible to obtain a correct time constant by doubling the ratio of sensory to motor variance (by either doubling Σ*_sens_* or halving Σ*_mot_*), while it is possible to obtain correct ACF(1)s for the Direction and Extent components by doubling the aspect ratio of Σ*_pl_* and halving that of Σ*_ex_*.

**Table 3:** Results of the sensitivity analysis for the Kalman filter for condition M.

	*w*	*s*	*c*	Time cst.	ACF(1)Ext	ACF(1)Dir
Observed				1.04	0.102	0.103
Baseline	0.45	17.6	2.57	0.65	0.032	0.158
Σ*_mot_* halved	0.22	14.1	2.80	0.82	0.062	0.190
Σ*_mot_* doubled	0.66	17.5	2.39	0.57	0.013	0.121
AR(Σ*_mot_*) halved	0.56	17.4	2.45	0.62	0.058	0.124
AR(Σ*_mot_*) doubled	0.30	19.1	2.78	0.71	0.008	0.178
Σ*_sens_* halved	0.63	17.5	2.36	0.58	0.011	0.115
Σ*_sens_* doubled	0.22	14.1	2.80	0.80	0.063	0.190
AR(Σ*_sens_*) halved	0.39	18.9	2.69	0.67	0.003	0.170
AR(Σ*_sens_*) doubled	0.49	16.5	2.58	0.65	0.063	0.130
AR(Σ*_pl_*) halvedAR(Σ*_ex_*) doubled	0.55	17.4	3.02	0.60	0.039	0.217
AR(Σ*_pl_*) doubledAR(Σ*_ex_*) halved	0.52	15.6	2.78	0.65	0.059	0.118
Σ*_pl_* isotropic	0.56	17.9	1.98	0.68	0.039	0.182

AR: aspect ratio.

**Table 4:** Results of the sensitivity analysis for the Kalman filter for condition L.

	*w*	*s*	*c*	Time cst.	ACF(1)Ext	ACF(1)Dir
Observed				1.29	0.314	0.295
Baseline	0.32	8.3	7.84	0.80	0.200	0.354
Σ*_mot_* halved	0.12	5.1	6.65	1.43	0.205	0.305
Σ*_mot_* doubled	0.73	13.9	3.61	0.59	0.147	0.340
AR(Σ*_mot_*) halved	0.38	7.6	4.61	0.85	0.255	0.335
AR(Σ*_mot_*)doubled	0.39	10.6	7.53	0.78	0.179	0.419
Σ*_sens_* halved	0.72	13.9	3.69	0.60	0.148	0.339
Σ*_sens_* doubled	0.10	3.6	8.80	1.55	0.219	0.318
AR(Σ*_sens_*) halved	0.50	12.9	4.21	0.74	0.148	0.410
AR(Σ*_sens_*)doubled	0.29	7.2	6.32	0.88	0.240	0.316
AR(Σ*_pl_*) halvedAR(Σ*_ex_*) doubled	0.76	15.0	4.46	0.62	0.241	0.427
AR(Σ*_pl_*) doubledAR(Σ*_ex_*) halved	0.36	6.0	5.63	0.94	0.283	0.260
Σ*_pl_* isotropic	0.71	15.9	2.91	0.72	0.236	0.414

AR: aspect ratio.

In summary, the sensitivity analysis demonstrates that the failure of the Kalman filter to explain the data cannot be the result of making incorrect assumptions about the underlying covariance matrices for conditions H and M, whereas this model can explain the results of condition L only if at least three parameters are changed to extreme values. Together, this suggests that the motor system does not use a Kalman filter to determine the learning rate in every individual movement.

### EPAPC Model

To obtain a better understanding of the actual learning rate, we compared the observed behaviour to the predictions of a second model. This model is almost the same as the Kalman filter, but rather than using the time-varying, optimal Kalman gain as learning rate, it uses a learning rate **B** that is the same for all movements in a condition, but that can vary between conditions:
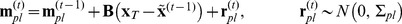
(15a)

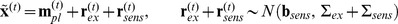
(15b)


The only difference between these equations and equation 11 of the Kalman filter is that the Kalman gain has been replaced by learning rate **B**. There are no state-estimation equations for this model because it does not perform state estimation – there is just a fixed learning rate for each level of error-signal reliability. Since this model is an extension of the Planned Aim Point Correction (PAPC) model used in [21], we will refer to it as the Extended Planned Aim Point Correction (EPAPC) model. The model is extended at two places: (1) it includes sensory noise, which is not included in the PAPC model, and (2) the learning rate is a matrix, whereas it is a scalar in the PAPC model. The inclusion of the sensory noise is straightforward; error corrections are driven by sensed rather than actual errors. The advantage of using a matrix rather than a scalar learning rate is that it allows us to test whether the learning rate is different for the Extent and Direction components. We assumed that the learning rate **B** is a diagonal matrix because all observed cross correlations were about zero.

### EPAPC Model: Predictions

We tested the EPAPC model in the same way as the Kalman filter: by examining whether it can reproduce the observed time constants and autocorrelations. The same parameters *w* and *s* as for the Kalman filter were used to parameterize Σ*_pl_* and Σ*_ex_*; *c* was not a free parameter here, but was in each simulation chosen such that it reproduced the observed initial value of the learning curve (the resulting time constant and ACF(1)s were virtually independent of this value in a wide neighbourhood around the value used). The diagonal elements of **B**, *B_ext_* and *B_dir_*, were the other free parameters. The total number of free parameters was therefore four (*w*, *s*, *B_ext_* and *B_dir_*), but, to prevent overfitting, this number was restricted to three for each condition. For condition H, *s* was not a free parameter as the time constant and autocorrelations were independent of this parameter. For conditions M and L, *w* was not a free parameter, but its value was chosen the same as estimated for condition H (0.197).

Table 5 shows the means (of all subjects) and standard errors of the best parameter estimates. The last column of Figure 3 shows the predictions of the EPAPC model, based on the mean (between subjects) of the parameter estimates. Like the Kalman filter, this model can explain that the time constant and ACF(1)s increase with decreasing error-signal reliability. In addition, and contrary to the Kalman filter, the EPAPC model can also explain the actual values of the time constants, as the predicted time constants were close to the observed ones (0.84 vs. 0.81, 0.95 vs. 1.04, and 1.33 vs. 1.29 movements, for condition H, M and L, respectively; all *p*>0.5, two-tailed weighted *t* tests: [26]). This model also reproduces the autocorrelations (compare Figs. 3F, L, R to Figs. 3D, J, P). Two-tailed *t* tests confirmed that none of the predicted ACF(1)s differed significantly from the observed values (all *p*>0.1), and that the predicted ACF(1) was not significantly different for the Extent and Direction components (*p*>0.25 for each condition).

**Table 5:** Best parameter estimates (means of all subjects ± s.e.m.) for the EPAPC model.

Condition	*w*	*s*	*B_ext_*	*B_dir_*
H	0.20±0.05	–	0.33±0.03	0.40±0.05
M	–	13.3±3.5	0.29±0.04	0.33±0.04
L	–	7.2±2.0	0.15±0.03	0.29±0.06

These results suggest that subjects used a fixed learning rate for all trials in an experimental condition. The estimated learning rates for the Extent and Direction component in condition H were 0.33±0.03 and 0.40±0.05 (mean of all subject ± standard error), respectively. These values were not significantly different (two-sided paired *t* test: *p* = 0.29), which implies that the scalar learning rate used in [21] was appropriate for this condition. For condition M, the learning rates were 0.29±0.04 and 0.33±0.04, which also did not differ significantly from each other (*p* = 0.48). For condition L, the learning rates were 0.15±0.03 and 0.29±0.06, which were significantly different (*p* = 0.006). Learning rates decreased with increasing error-signal uncertainty (see Fig. 5C).

We conducted a sensitivity analysis to determine how the estimates of the learning rates depend on the assumptions made regarding the covariance matrices. We varied the same parameters as for the sensitivity analysis of the Kalman filter. For conditions M and L we also halved and doubled the assumed value of *w*. Tables 6, 7 and 8 show that the effects of all these variations on the estimated learning rates are relatively small. In particular, the dependence of the learning rates on the error-signal reliability and on the component considered (Extent vs. Direction) remains the same in all cases considered.

**Table 6:** Results of the sensitivity analysis for the EPAPC model for condition H.

	*B_ext_*	*B_dir_*	*w*	Time cst.	ACF(1)Ext	ACF(1)Dir
Observed				0.81	0.002	−0.050
Baseline	0.33	0.40	0.197	0.83	0.005	−0.049
Σ*_mot_* halved	0.33	0.40	0.195	0.85	0.004	−0.048
Σ*_mot_* doubled	0.33	0.40	0.195	0.84	0.006	−0.050
AR(Σ*_mot_*) halved	0.33	0.40	0.195	0.84	0.006	−0.050
AR(Σ*_mot_*)doubled	0.33	0.40	0.195	0.84	0.005	−0.049
AR(Σ*_pl_*) halvedAR(Σ*_ex_*) doubled	0.27	0.50	0.155	0.79	−0.006	−0.026
AR(Σ*_pl_*) doubledAR(Σ*_ex_*) halved	0.42	0.32	0.252	0.83	0.015	−0.063
Σ*_pl_* isotropic	0.29	0.53	0.369	0.82	−0.017	−0.042

For all of these simulations, we assumed *s* = 1.

AR: aspect ratio.

**Table 7:** Results of the sensitivity analysis for the EPAPC model for condition M.

	*B_ext_*	*B_dir_*	*s*	Time cst.	ACF(1)Ext	ACF(1)Dir
Observed				1.04	0.102	0.103
Baseline	0.29	0.33	13.3	0.95	0.058	0.084
*w* halved	0.21	0.25	10.9	1.21	0.039	0.066
*w* doubled	0.41	0.43	19.1	0.75	0.089	0.111
AR(Σ*_mot_*) halved	0.34	0.33	10.6	0.89	0.029	0.066
AR(Σ*_mot_*)doubled	0.27	0.34	17.0	0.96	0.073	0.104
Σ*_sens_* halved	0.28	0.31	10.6	1.00	0.060	0.069
Σ*_sens_* doubled	0.30	0.35	16.5	0.91	0.061	0.108
AR(Σ*_sens_*) halved	0.27	0.34	14.9	0.97	0.071	0.091
AR(Σ*_sens_*)doubled	0.30	0.29	12.1	0.97	0.058	0.087
AR(Σ*_pl_*) halvedAR(Σ*_ex_*) doubled	0.24	0.49	15.5	0.86	0.063	0.117
AR(Σ*_pl_*) doubledAR(Σ*_ex_*) halved	0.34	0.20	11.9	0.99	0.060	0.043
Σ*_pl_* isotropic	0.24	0.37	10.2	1.01	−0.016	0.081

AR: aspect ratio.

**Table 8:** Results of the sensitivity analysis for the EPAPC model for condition L.

	*B_ext_*	*B_dir_*	*s*	Time cst.	ACF(1)Ext	ACF(1)Dir
Observed				1.29	0.314	0.295
Baseline	0.15	0.29	7.2	1.32	0.249	0.293
*w* halved	0.17	0.36	3.1	1.25	0.196	0.359
*w* doubled	0.20	0.28	18.8	1.21	0.317	0.298
AR(Σ*_mot_*) halved	0.17	0.27	5.6	1.30	0.249	0.261
AR(Σ*_mot_*)doubled	0.15	0.29	10.5	1.36	0.242	0.319
Σ*_sens_* halved	0.15	0.27	6.0	1.35	0.234	0.234
Σ*_sens_* doubled	0.15	0.28	11.6	1.38	0.255	0.321
AR(Σ*_sens_*) halved	0.15	0.29	8.4	1.37	0.237	0.308
AR(Σ*_sens_*)doubled	0.17	0.28	5.8	1.28	0.247	0.269
AR(Σ*_pl_*) halvedAR(Σ*_ex_*) doubled	0.14	0.34	15.8	1.25	0.209	0.308
AR(Σ*_pl_*) doubledAR(Σ*_ex_*) halved	0.21	0.26	2.9	1.20	0.298	0.324
Σ*_pl_* isotropic	0.23	0.41	3.2	1.08	0.176	0.352

AR: aspect ratio.

We will now look in more detail at some other aspects of the observed learning and compare these to the predictions of the EPAPC model. Figure 3 shows that the model reproduces also the final part of the learning curves, i.e., the flat end in conditions H and M, and the rising end in condition L. This is related to the size of the autocorrelations and to using the Mahalanobis distance to construct learning curves. For ACF(1)s close to zero (conditions H and M), consecutive endpoints are (close to) independent of one another. The mean Mahalanobis distance will therefore be the same for all but the first few movements in a series, leading to a flat last part of the learning curve. In contrast, for ACF(1)s substantially greater than zero (condition L), endpoints of consecutive movements are relatively close together. The endpoints within a series will therefore ‘drift’, somewhat like a random walk. As a result, the first and last endpoint of a series will on average be further away from the mean than endpoints in the middle of the series. The expected Mahalanobis curve is therefore U-shaped, where the large errors in the beginning of the series make it asymmetric. This effect explains the rising end of the learning curve in condition L.

Figures 6A, D, G show that in all conditions the observed partial autocorrelations (PACFs) are close to zero for lags 2 and 3, while they tend to be negative for larger lags. Figures 6B, E, H show that the PACFs predicted by the model follow a similar pattern. This indicates that the model also reproduces the longer-range interactions between movements in a series. It is however surprising that the PACFs at large lags are negative, because the model includes corrections based on the error in only the previous movement, not in movements longer ago. The negative PACFs could be an artefact of estimating them from relatively short time series [19], [20]. To examine whether this is the case, we simulated the model also for hypothetical experiments with series of 1,000 movements. The PACFs predicted for these long series (Figs. 6C, F, I), which can be considered as close approximations of the ‘true’ PACFs, approach zero, which confirms that the negative PACFs at lags greater than 3 are an artefact of using short time series.

**Figure 7:**
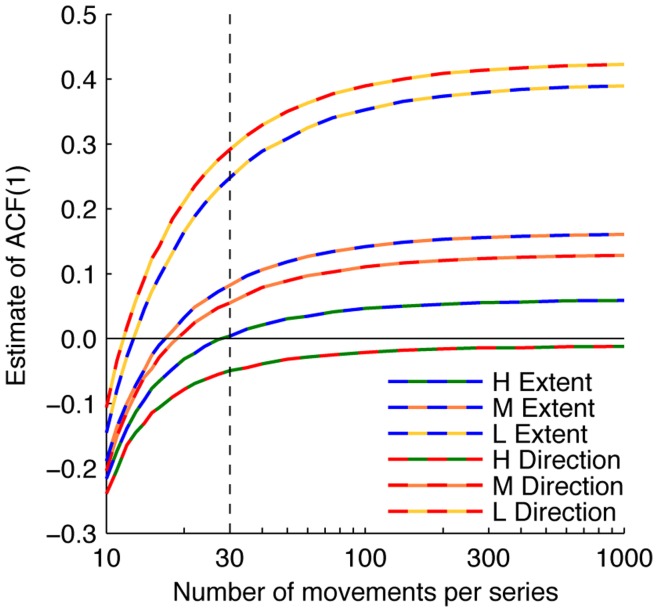
Estimated lag 1 autocorrelations as a function of the series length. The curves represent the estimated ACF(1) of the Extent and Direction components, as found by simulations for a range of series lengths for each experimental condition. The black dashed line indicates the series length used in the experiment (30).

A comparison of the middle and right columns of Figure 6 reveals that the autocorrelations at lag 1 also depend on the series length. Short time series lead to underestimates of the PACF(1) in all conditions. Since at lag 1, PACFs are equal to ACFs, this suggests that all ACF(1)s in Figure 3 represent underestimates of the actual ACF(1). To see how the estimated ACF(1) depends on the number of movements in a series and on the error-signal uncertainty, we simulated the model for hypothetical experiments with series in the range of 10 to 1,000 movements (see Fig. 7). The estimated ACF(1) increases with error uncertainty and with series length. For the series of 30 movements used here (black dashed line in Fig. 7), all curves are still rising considerably. Thus, different estimates of the ACF(1)s would have been found if shorter or longer series had been used. Figure 7 thus highlights the importance of taking the series length into account when interpreting observed autocorrelations. We stress that this effect does not reflect different error correction behaviour for different series lengths; it only reflects series-length dependent biases in the estimation of autocorrelations. Note that this is not a problem for testing the models, as ACF(1)s of both real and simulated data are subject to the same bias.

### Optimality of the Learning Rates

**Figure 8:**
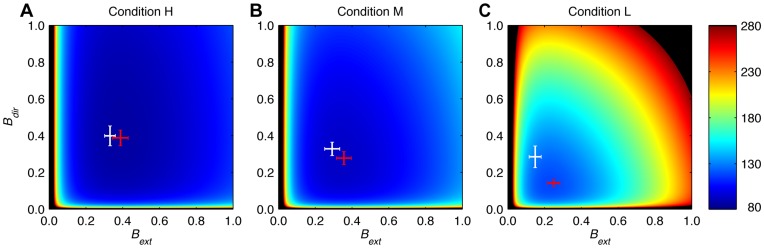
Endpoint variance (mm^2^) as a function of the learning rates according to the EPAPC model. Endpoint variance (Eq. 28) is plotted as a function of the learning rates in the Extent and Direction component. The best estimates (mean of all subjects) of the model parameter were used to generate these plots. The value of *s* that was estimated for condition M was used for all conditions. Variances exceeding 280 mm^2^ are shown in black. White: observed learning rates. Error bars represent standard errors. Red: the optimal learning rates that minimize the variance (Eq. 29). The error bars herein were determined from the uncertainty in the parameter estimates. **A** Condition H. **B** Condition M. **C** Condition L.

Although we found that the learning rate was not optimal for each individual movement (i.e., the data are not consistent with the Kalman filter), it is possible that learning rates were optimal under the restriction that they were the same for all trials of the same condition, as in the EPAPC model. With optimal, we mean that they had the value that minimized the endpoint variance. To examine whether this was the case, we derived an expression for the endpoint variance Var(**x**) as predicted by this model (see Methods):
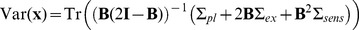
(16)where Tr denotes the matrix trace. This equation can be written as the sum of variances of the Extent and Direction components (see Eq. 27 in Methods). Figure 8 shows, for each condition, the predicted variance as a function of the learning rates in these two dimensions. The variance differs between conditions, but for each condition the variance is large for very small learning rates of each component, it increases also for large values and it reaches a minimum for intermediate values. For very small learning rates, corrections are too small, so that changes of the planned aim point are mainly driven by planning noise, leading to a large variance. For large learning rates, corrections are too large, overshooting the target, also giving rise to a large variance. For intermediate learning rates, the deleterious effects of small and large learning rates cancel, resulting in a smaller variance. The positions of the minima can be found in closed form (see Eq. 29 in Methods), and are indicated in Figure 8 in red. The optimal learning rate decreases with increasing error uncertainty for both components. For zero error uncertainty (condition H), the optimum occurs for the learning rate for which the autocorrelation vanishes [21]. If there is finite error uncertainty (conditions M and L), the variance of the *sensed* endpoints is minimal for the learning rate for which the autocorrelation of the *sensed* endpoints vanishes. This is also the learning rate that minimizes the variance of the actual endpoints (this variance is a fixed amount Tr(Σ*_sens_*) smaller than that of the sensed endpoints). The autocorrelation of the *actual* endpoints is positive for this learning rate.

The learning rates estimated from the data (indicated in Figure 8 in white) are close to the optimal ones in conditions H and M, for both the Extent and Direction component. In contrast, the learning rates of both components differed from the optimal ones in condition L. Whereas the EPAPC model (and also the Kalman filter, see Fig. 3N) predicts a larger learning rate for the Extent than for the Direction component, the data suggest the converse.

It is surprising that learning rates were near-optimal in two conditions, but not in the third. This could be a result of drift in the proprioceptive sense of hand location in condition L. Although care was taken to minimize such drift by giving subjects visual feedback about their finger location at the beginning of each trial [27], there could have been some drift in the felt hand location at the end of the movements. Drift leads to a positive autocorrelation, and therefore to an underestimate of the learning rate. This could explain why the observed *B_ext_* (0.15) was smaller than the optimal one (0.23). To check whether this was the case, we regressed for each series the Extent and Direction components of the endpoints against the trial number. We made use of the finding that proprioceptive drift has, per subject, a fixed direction [27], [28] to separate genuine drift from the effects of random endpoint variations that can also lead to non-zero regression slopes. To this end, we fitted for each subject the regression slopes *β* for the 24 target directions *θ* to the function *β*(*θ*) =  *p*_1_ *+ p*_2_ sin(*θ*) + *p*_3_ cos(*θ*). Here, *p*_1_ represents drift that all series have in common, such as an increasing Extent, whereas *p*_2_ and *p*_3_ account for drift that is constant in external space, such as a rightward drift. The drift captured by this fit was subtracted from the actual endpoints, after which the whole analysis was repeated. This led to estimated learning rates of 0.16±0.03 and 0.30±0.06 for Extent and Direction, respectively. These values differ only marginally from the ones obtained without drift correction, and they differ substantially from the optimal ones. This demonstrates that proprioceptive drift cannot explain the difference between observed and optimal learning rates in condition L.

## Discussion

We analyzed the time-series statistics of repeated reaching movements with different levels of error-signal reliability to determine the learning rate used by the motor system for updating motor planning on the basis of observed errors. We found that the learning rate increases with increasing error-signal reliability, which agrees with the results of earlier studies [11], [12] and with the predictions of the Kalman filter. However, the Kalman filter cannot explain the learning rate of every individual movement, because learning at the beginning of a series was not as fast as predicted by this model, and the learning rate of the Extent and Direction components did not differ in the predicted way. In contrast, the data were consistent with the EPAPC model, in which the learning rate is fixed for all movements with the same error-signal reliability. Moreover, these fixed learning rates were optimal for minimizing the endpoint variance for two levels of error-signal reliability but not for the lowest.

One could argue that the comparison between the Kalman filter and the EPAPC model is not fair because the EPAPC model had one free parameter more. However, we parameterized the Kalman filter such that it had the highest possible number of free parameters (three): The variance of the process noise, of the measurement noise and of the initial state estimate fully determine the time constant of the learning curve and the steady-state lag 1 autocorrelation. The fact that this model is unable to reproduce both the time constant and the autocorrelation simultaneously demonstrates that subjects cannot have used a Kalman filter to determine the learning rate in every individual trial. This is confirmed by the sensitivity analysis, as this analysis showed that the Kalman filter is also unable to reproduce the data when the underlying variances are varied by large amounts. Extending the Kalman filter from a single-state to a two-state model in which the two states have different learning and retention rates [29] cannot solve these problems either because single-state and two-state models are identical in the absence of perturbations [21]. Since the Kalman filter is the optimal state estimator, we conclude that subjects did not choose learning rates that would produce the smallest possible mean squared endpoint error for every movement. Instead, their performance was well modelled by the EPAPC model. This model differs from the Kalman filter in two respects: it uses a fixed learning rate (for a given level of error-signal reliability), and it does not automatically optimize the learning rate. We will now discuss these differences.

We first emphasize that we cannot exclude that learning rates were not completely fixed per condition. They could have decreased at the beginning of a series, but to a smaller extent than predicted by the Kalman filter. Wei and Körding [12] varied the uncertainty of the system’s state estimate prior to a perturbation, and found that the magnitude of the resulting correction increased with increasing state uncertainty, but this effect was small. This suggests that in our experiment the learning rate may have decreased slightly at the beginning of the series, but less than predicted by the Kalman filter. A constant learning rate will therefore be a good approximation. Why would the brain prefer an approximately constant learning rate to a flexible one that produces a smaller endpoint variance? An obvious advantage is that the brain does not need to do sophisticated state estimation and compute the learning rate for every individual movement. Consistent with this, Wei et al. [30] found that error-driven planning corrections are nonspecific as they are the same for perturbations of different natures (i.e., visual disturbances, forces acting on the arm or changes of the inertia). Another advantage of a fixed learning rate is that it is robust to incorrect assumptions about the origin of an observed error. If one would determine the optimal learning rate in every trial and, for instance, observe a large error and assume this was due to incorrect planning, one would make a large correction [13], [14]. If, however, the error had an external origin that was transient, the large correction would be inappropriate and would result in another large error. This will happen to a lesser extent when a fixed learning rate is used.

A next question is why our motor system chooses the specific learning rate that is chooses for a given error-signal reliability. It may be chosen to minimize the endpoint variance as it was close to the, for this purpose, optimal value in two of the three conditions. However, it was not optimal for the condition with the lowest error-signal reliability. We showed that this difference cannot be the result of proprioceptive drift, and the sensitivity analysis suggests that it cannot be explained by incorrect assumptions about model parameters either. Why did the learning rate differ from the optimal value in this condition? One possibility is that subjects used an incorrect estimate of the error-signal reliability, and used a learning rate that was optimal for this incorrect value. Using Eq. 29, one can show that this would mean that subjects overestimated the error-signal variance of the Extent component by more than a factor 2, and underestimated that of the Direction component by more than a factor 10. It is unlikely that subjects misestimated these variances by such large amounts as studies on visual-proprioceptive integration [31]–[33] and sensorimotor adaptation [34], [35] suggest that the sensorimotor system has accurate knowledge of the precision of vision and proprioception, and even their anisotropy. Another possibility is that the non-optimality of the learning rates in the condition with the lowest error-signal reliability is related to the fact that this condition is qualitatively different than the other two conditions, as subjects received no visual feedback about their errors in this condition. Receiving no visual feedback is different from receiving highly unreliable visual feedback. The underlying generative models are different, and even though the two could under certain conditions be mathematically equivalent, it is unclear whether these conditions are fulfilled in the brain. Future research is required to examine whether this can explain the results of the condition with the lowest error-signal reliability.

We found that the learning rate was the same for the Extent and Direction components for conditions H and M. This confirms that the scalar learning rate that was used in the model of van Beers [21] for condition H was appropriate. In contrast, the learning rate of the Direction component was larger than that of the Extent component in condition L. This is surprising for three reasons. First, the resulting endpoint variance would be smaller if it was the converse (Fig. 8C). The Kalman filter also predicts a larger learning rate for the Extent than for the Direction component (see inset of Fig. 3N). This is because the ratio of measurement to process noise variance is larger for the Extent than for the Direction component. Second, Burge et al. [11] tested whether the learning rate changes when the ratio of measurement to process noise variance is changed. They varied this ratio by making the measurement noise anisotropic while keeping the process noise the same, and found a change in the direction that minimizes the endpoint variance. Third, visuomotor adaptation studies [36], [37] found that adaptation to a gain change is faster than adaptation to a rotation, which also corresponds to a larger learning rate for the Extent than for the Direction component. All these findings are inconsistent with our result. A possible explanation is that our brain takes the anisotropy of the error signals into account when determining the learning rate, but ignores the anisotropy of the endpoints distribution. Another possibility is that learning rates following large errors that are attributed to an external origin, as may happen in experiments with perturbations, are different than learning rates following smaller errors that are self-generated. Future research is required to test these ideas.

Our main conclusion is that our brain does not determine the optimal learning rate for every individual movement, as a Kalman filter would do. Instead, the learning rate is approximately the same for all movements with the same level of error-signal reliability. The average behaviour is thus near optimal, but individual movements are not optimal. This conclusion applies to the particular case of determining the learning rate in motor learning, but the issue is relevant to many other cases in the sensorimotor domain as well. Examples include feedback control of on ongoing movement, integration of sensory information and bimanual coordination. Since neural algorithms that produce behaviour that is optimal for every individual movement may be different, probably more complicated, from algorithms that produce behaviour that is only optimal on average, much insight into our motor system can be gained from addressing the issue for other sensorimotor tasks. The present results suggest that our motor system may not try to optimize individual movements but cares more about the average behaviour.

## Methods

### Experimental Methods

#### Subjects

Eight subjects (three female, five male, 18–24 years old) participated in all experimental conditions. None of them reported any sensory or motor deficits, and all had normal or corrected-to-normal vision, reported being right handed, and were unaware of the purposes of the study.

#### Ethics statement

All subjects gave verbal informed consent (which was then documented) before participation. All experiments were conducted in agreement with the ethics and safety guidelines of the Science Faculty of Utrecht University, where the experiment was conducted, and was part of a program that received blanket approval of the Medical Ethical Test Committee of the University Medical Centre Utrecht. All data were encoded and analyzed anonymously.

#### Apparatus

The same set up was used as in [21]. Subjects were seated at a table (98 cm wide and 55 cm deep) on a height-adjustable chair. They looked down in a horizontal mirror that was mounted above the tabletop and they saw images that were projected on a projection screen by an LCD projector (1280×720 pixels, 60 Hz) (Fig. 1A). The mirror was placed midway between the tabletop and the projection screen so that it looked as if the projected images appeared on the tabletop. An Optotrak Certus system (Northern Digital, Waterloo, Ontario) recorded the position of an infrared emitting diode that was attached to the nail of the right index finger (300 Hz, 2D accuracy: better than 0.1 mm). Subjects could not see their arms because these were hidden by the mirror and a black cloth that was draped over the shoulders.

#### Procedure

The task was to move the tip of the right index finger from a start position to visual targets. The start position was a pink disc (4 mm radius) at a fixed location approximately 35 cm in front of the waist. A red cursor (a 4 mm radius disc) was shown at the fingertip location when it was within 3 cm from the start position. This enabled subjects to place their finger quickly and accurately on the start position, and it also prevented drift of the perceived finger location throughout an experimental session [27]. When the finger had been within 0.5 cm from the start position for 1 s, the finger cursor turned green, and a yellow target (a 4 mm radius disc) appeared. The instruction was to make a quick, uncorrected movement to the target. The finger cursor went off when the finger speed exceeded 2 cm/s. At this moment, the finger had usually moved less than 1 mm, so that subjects received no informative visual feedback about their movement trajectory. The movement endpoint was determined online as the location where the finger speed first fell below 2 cm/s, and it was displayed immediately. The way in which it was displayed depended on the experimental condition:

Condition H (high error reliability): A red disc (4 mm radius) was shown at the endpoint location. It was shown alongside the target so that the error signal was highly reliable. A score was awarded based on the distance from the target (see Fig. 1B). This condition was identical to Experiment 1 in [21], and the data of this condition presented here are the same as in that paper.

Condition M (medium error reliability): A cloud of 15 red circular dots (0.8 mm radius) was shown. The dot locations were drawn independently from a circular Gaussian distribution with the actual endpoint as the mean and a standard deviation of 15 mm (see Fig. 1C). New dot positions were generated in every trial. No score was awarded in this condition.

Condition L (low error reliability): Subjects received no visual feedback about their movement endpoints (see Fig. 1D), but they could compare the proprioceptively felt finger position to the seen target location.

In all conditions, the visual feedback, if any, was shown for 1 s. After that, subjects moved their finger back to the start position to begin the next trial.

A session consisted of 24 series of 30 movements each, all in the same experimental condition. The targets were located at 10 cm distance from the start position in equally spaced directions. A blocked design was used, in which the same target was used for all movements in a series. The target of the first series was randomly chosen exactly to the left of right of the start location. Each later target direction differed 105 degrees from the previous direction in the counter clockwise direction. There were breaks of at least 10 seconds between series. At the start of a session, each subject practiced the task in the condition of that session for several minutes before starting the experiment proper (with a different target than in the first series). A session lasted approximately one hour. Each subject performed one session of each condition, each on a different day. The order of conditions was randomized between subjects.

#### Analysis

We analyzed the two-dimensional movement endpoints. A small fraction of the movements (0.56%, 0.42% and 0.21% in conditions H, M and L, respectively) was discarded from the analysis because the recording had failed. Endpoints were transformed into an Extent component (the component parallel to the vector from the start location to the mean endpoint of the series) and a Direction component (the component orthogonal to the Extent component). To characterize error-corrective learning, we determined two measures: Mahalanobis distance and serial correlations.

The Mahalanobis distance was calculated to construct learning curves. At first sight, a plot of the mean error magnitude as a function of the movement number in the series could serve as a suitable learning curve. However, since endpoint distributions were anisotropic (Fig. 2), such a curve would mainly reflect changes in movement extent and practically ignore changes in movement direction. Moreover, in condition L the endpoint often shifted away from rather than towards the target (see Fig. 2C for examples). These are two reasons why the mean error magnitude is not a suitable measure to construct learning curves. The Mahalanobis distance does not suffer from these problems. We calculated Mahalanobis distance *D* ^(*t*)^ of movement *t* in a series as:
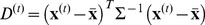
(17)


where **x** ^(*t*)^ is the endpoint of movement *t*, 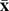 and Σ are the mean and covariance matrix of all endpoints in the series, and *T* denotes the matrix transpose. The Mahalanobis distance can be interpreted as the squared number of standard deviations that a given endpoint differs from the mean endpoint in its series. It takes the anisotropy into account and weights the Extent and Direction components equally. Since it is a normalized quantity, it can be averaged across series and subjects, even when their variance differs.

A learning curve was constructed for each subject by calculating the Mahalanobis distance of each endpoint, and then averaging these across series as a function of the trial number in the series. Time constants of the learning curves, and their 95% confidence intervals, were estimated for individual subjects using nonlinear least-squares regression. We fit exponentials of the form *a* + *b*(1– exp(–*t*/*t_c_*)) to the learning curves, where *t* is the movement number, *a* and *b* are constants and *t_c_* is the time constant. Since the time constant could occasionally not be determined reliably, as indicated by a large confidence interval, a weighted average over subjects was calculated by weighing each subject’s time constant by the inverse of the squared width of the confidence interval.

Whereas learning curves are informative about correction for large errors in the beginning of a series, the serial correlations focus on error correction in the “steady state” when errors are small. Serial correlations were calculated from the last 25 endpoints of each series, to avoid them being influenced by the correction for the large initial errors. Serial correlations express the statistical relationship between the endpoints of movements separated by a certain lag (number of movements) *k*. Since the endpoints are two-dimensional, the serial correlations consist of two autocorrelation functions ACF(*k*), one for each component (Extent and Direction), and two cross-correlation functions CCF(*k*) between the components. The cross-correlation function CCF*_i_*_,*j*_(*k*) between components *i* and *j* at lag *k* was calculated as:
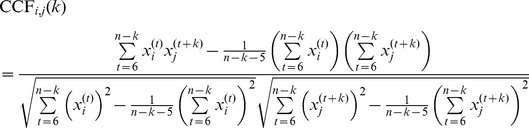
(18)where *x_i_* ^(*t*)^ denotes component *i* of the endpoint of movement *t*, and *n* is the number of movements in a series (30). Summations start at 6 because the first 5 movements were not included. The method developed by Marshall [38] was used to deal with missing values. The autocorrelation function ACF*_i_*(*k*) of component *i* at lag *k* was found as: ACF*_i_*(*k*) =  CCF*_i_*_,*i*_(*k*).

### Modelling

#### Model simulations

Two models for trial-by-trial motor learning are described in the Results section. We ran Monte Carlo simulations to determine the predictions of each model. Each simulation consisted of 2,000 sets of 24 simulated series of 30 movements, corresponding to 2,000 subjects performing a full experiment. Random vectors were drawn from Gaussian distributions to simulate the effects of planning, execution and sensory noise, as specified in the Results section.

For condition H, the sensory-noise covariance matrix Σ*_sens_* was assumed to be zero because subjects received highly reliable error signals. All subjects participated in a control condition to estimate Σ*_sens_* for condition M. This condition was similar to condition M, but now they saw the cloud of dots from the start of the movement and they moved their finger until they perceived it aligned with the target. Eight targets in equally spaced directions were tested. The variability in the indicated positions hardly varied between targets and subjects. Based on these results, we assumed that Σ*_sens_* was [17.4 0.0; 0.0 17.4] mm^2^ for each target. For condition L, we assumed that Σ*_sens_* equalled [62.5 0.0; 0.0 62.5] mm^2^, which is the sum of the variances of visual and proprioceptive (right hand) localization reported by van Beers et al. [39].

#### Fitting the models

The potentially most powerful method to estimate the parameters of a linear dynamic system from time-series data is maximum likelihood estimation using the expectation-maximization algorithm [40]. However, this method could not be used here as simulations showed that it produces biased estimates for the short time series used here. Instead, we fitted the models by finding the parameter values that best reproduced the observed learning curves and autocorrelations. For the Kalman filter, this amounted to estimating three parameters: *w*, *s* and *c* (but *s* was not estimated for condition H, see Results). For the EPAPC model, there were four free parameters: *w*, *s*, *B_ext_* and *B_dir_*, but only three of these were estimated per condition (see Results).

To find the best parameter estimates, we ran simulations for a range of parameter values. For instance, to fit the EPAPC model to the data of condition H, *B_ext_* and *B_dir_* were varied between 0.1 and 0.9, and *w* between 0.0 and 0.6. Tensor product splines were then fit (function spap2 in Matlab) to the resulting ACF(1)s, ACF(2)s and time constant as a function of *B_ext_*, *B_dir_* and *w*. Best parameter values were determined per subject by finding the values for which the sum of squares of the normalized difference between observed and predicted values of the time constant, and the ACF(1)s and ACF(2)s of Extent and Direction, was minimized. The differences were normalized by dividing each difference by the width of the confidence interval of the observed value. There is some arbitrariness in treating these five values equally. We verified that the parameter estimates hardly changed when the five values were weighted differently (such as giving the time constant a weight of 50% and the ACFs weights of 12.5%).

The best estimates of the parameter values for the Kalman filter are given in Table 1, and those of the EPAPC model in Table 5. One could be surprised by the different values of the estimate of *s* for conditions M and L. This probably reflects differential scaling of the assumed sensory-noise covariance matrices in these conditions. Indeed, whereas the matrix for condition M was estimated in a control condition, the matrix for condition L was estimated from literature values. Free parameter *s* was included exactly to account for such effects.

### EPAPC Model: Equations for Endpoint Variance and Autocorrelation

We use Equations 1 and 15 to find an expression for the endpoint in movement *t* +1 as a function of the endpoint in movement *t* and the various types of noise:
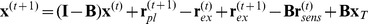
(19)


The expected endpoint is **x** *_T_* − **b** *_sens_*, where **b** *_sens_* is the bias in the sensory information (see “Kalman filter: model” in Results section). Define deviation **d** ^(*t*)^ in movement *t* as the difference between the endpoint and the expected endpoint:
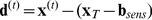
(20)


Then we have from (19):
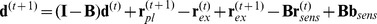
(21)


The covariance matrix function [41] of the deviations then is:
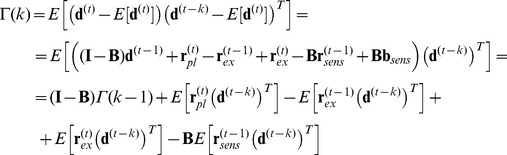
(22)where *E* denotes the expected value. After substitution of (21) and some algebraic manipulations, we find for *k* = 0:
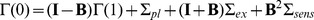
(23)and for k = 1 we find:



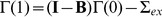
(24)The system of equations (23) and (24) has solution:
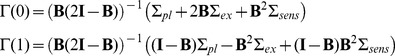
(25)


Γ(0) is the covariance matrix of the deviations. Since the deviations differ a constant vector from the endpoints, it is also the covariance matrix of the endpoints. When we define the endpoint variance Var(**x**) as the trace of the covariance matrix of the endpoints, we find equation (16). The lag 1 autocorrelation of component *i* (1 or 2, denoting Extent and Direction, respectively) is:
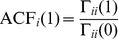
(26)


Note that the variance and autocorrelations are independent of sensory bias **b** *_sens_*.

The fact that all matrices in the above equations are diagonal (see Results) implies that the Extent and Direction components of the endpoints evolve independently of one another. The endpoint variance can therefore be written as the sum of an Extent and a Direction variance:
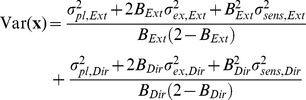
(27)where σ^2^ denotes variance. This equation can also be expressed in terms of the free parameters of the model:



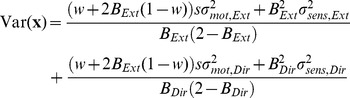
(28)The optimal learning rates can be found by finding the values of *B_ext_* and *B_dir_* for which the derivatives of the variance with respect to *B_ext_* and *B_dir_* are zero. These optimal learning rates are:
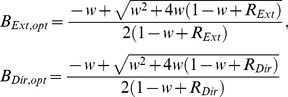
(29)


where: 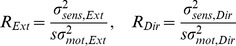.
